# Ecological Niche Modeling: An Introduction for Veterinarians and Epidemiologists

**DOI:** 10.3389/fvets.2020.519059

**Published:** 2020-10-21

**Authors:** Luis E. Escobar

**Affiliations:** Department of Fish and Wildlife Conservation, Virginia Polytechnic Institute and State University, Blacksburg, VA, United States

**Keywords:** spatial epidemiology, ecological niche modeling (ENM), disease mapping, ecological niche, distributional ecology

## Abstract

Most infectious diseases in animals are not distributed randomly. Instead, diseases in livestock and wildlife are predictable in terms of the geography, time, and species affected. Ecological niche modeling approaches have been crucial to the advancement of our understanding of diversity and diseases distributions. This contribution is an introductory overview to the field of distributional ecology, with emphasis on its application for spatial epidemiology. A new, revised modeling framework is proposed for more detailed and replicable models that account for both the biology of the disease to be modeled and the uncertainty of the data available. Considering that most disease systems need at least two organisms interacting (i.e., host and pathogen), biotic interactions lie at the core of the pathogen's ecological niche. As a result, neglecting interacting organisms in pathogen dynamics (e.g., maintenance, reproduction, and transmission) may limit efforts to forecast disease distributions in veterinary epidemiology. Although limitations of ecological niche modeling are noted, it is clear that the application and value of ecological niche modeling to epidemiology will increase in the future. Potential research lines include the examination of the effects of biotic variables on model performance, assessments of protocols for model calibration in disease systems, and new tools and metrics for robust model evaluation. Epidemiologists aiming to employ ecological niche modeling theory and methods to reconstruct and forecast epidemics should familiarize themselves with ecological literature and must consider multidisciplinary collaborations including veterinarians to develop biologically sound, statistically robust analyses. This review attempts to increase the use of tools from ecology in disease mapping.

## Introduction

Spatial epidemiology is the branch of epidemiology that aims to understand the geographic distribution of diseases (including its causative agents, hosts, and related factors) (1, 2). Most diseases in animals are not distributed randomly across landscapes or regions. Instead, researchers can quantitatively determine specific environmental factors associated with the occurrence of disease (3, 4). Reports of the spatial location of pathogens, disease vectors, or reservoirs are becoming more abundant, high quality, and openly accessible for a series of infectious diseases. Similarly, data on environmental variables are increasing in availability and cover diverse spatial and temporal scales: from meters to continents and from days to centuries (both retrospective and predictive). For example, many datasets of soil composition and structure (5), landscape composition and structure (6), and climate and geomorphology (7–9) are freely and openly available for mapping diseases in aquatic and terrestrial ecosystems globally. These variables can be linked with disease data to reconstruct or predict the geographic distribution of environmentally (e.g., anthrax), vector-borne (e.g., Bluetongue disease), and directly transmitted diseases (e.g., rabies), which are important to veterinary medicine. These studies, however, need a basic understanding of Geographic Information Systems, spatial statistics, and a deep understanding of the biology of the disease system to be modeled.

Disease models accounting for environmental information are particularly informative to understand the spread of diseases that are undergoing range expansion, which is termed “distributional disequilibrium” in ecology (10–12). Ecological theories and methods are commonly used in spatial epidemiology to design and interpret models of conditions where infections are likely to occur, with outputs projected to geography as measures of “suitability.” Suitability has been defined as the “sum” of the effects of resource and environmental conditions on the fecundity, demography, and survivorship of populations (13). Ecological niche modeling has been the main branch of ecology employed to map disease transmission. Comprehensive reviews are available elsewhere regarding the fundamentals of ecological niche modeling for epidemiologists interested in its applications on medical geography of infectious diseases (14–16). This manuscript is an overview of the field of ecological niche modeling for veterinarians and epidemiologists and considers parasites (e.g., tapeworm) and pathogens (e.g., virus) as agents causing disease. The content of this review is a friendly introduction to more specialized literature and study cases described in more detail elsewhere (17, 18).

### Models

A model is a simplification of a complex system. For example, in biomedicine, mice could be used as animal models to understand the effects of a drug in humans. In mathematics and statistics, equations can be used to simplify and summarize complex phenomena. Some mathematical models can be complex, by accounting for many details (i.e., parameters) in the disease system, while other models can be simple, accounting for just a few, key components of the system. Models can be used to reconstruct the structure or functioning of the system in question—termed descriptive models (e.g., the specific temperatures where a disease vector is found). Complementarily, models could be used to anticipate how the system would respond to determined “what-if-scenarios” —termed predictive models (e.g., the expected distribution of a disease vector under future temperature). Descriptive models are the basis and first stage for the development of predictive models.

**Figure 1:**
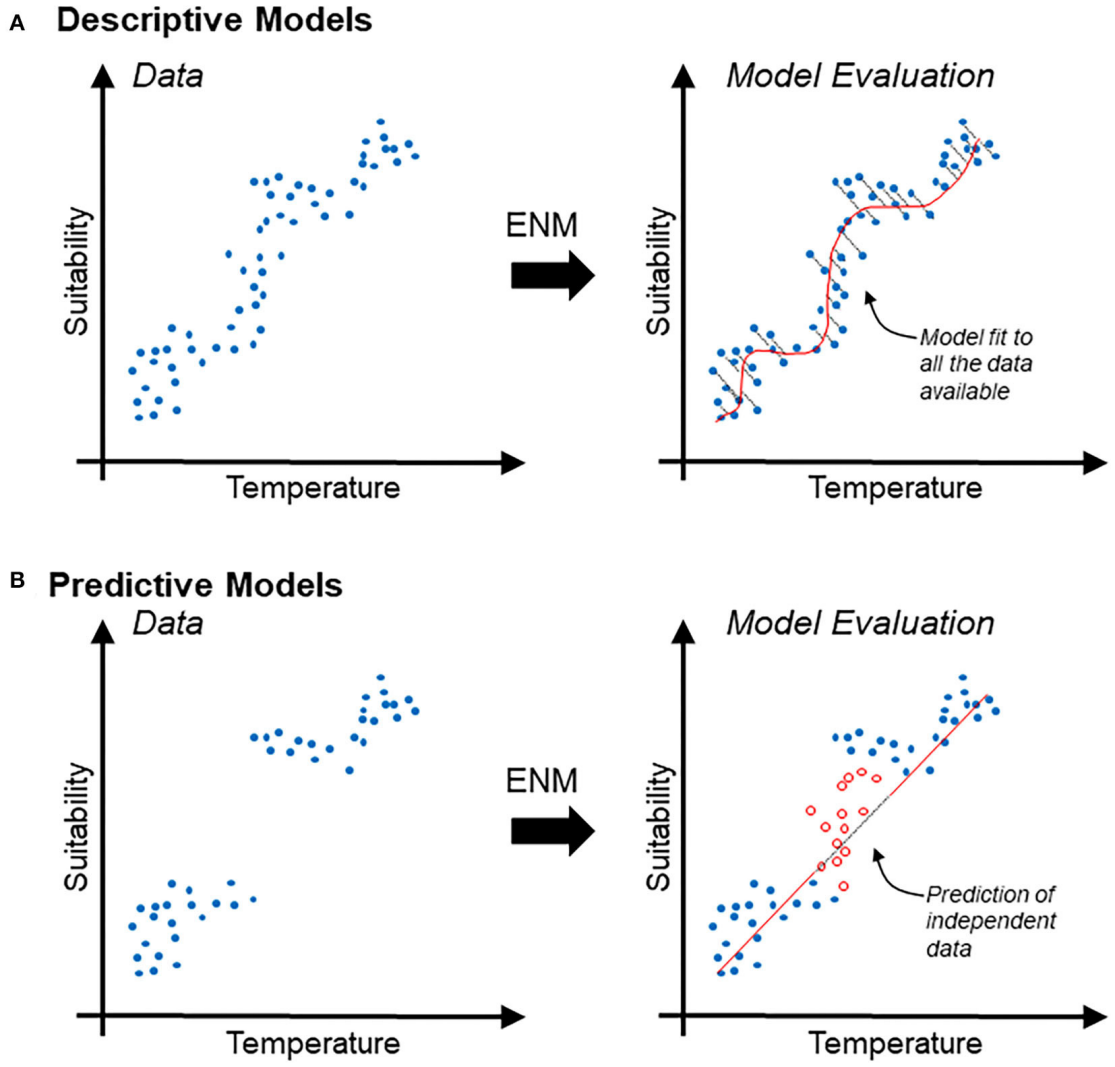
Schematic description of descriptive vs. predictive models in the context of ecological niche modeling. **(A)** Disease occurrence data (blue points) used to estimate relationships between temperature (independent variable; *x*-axis) and suitability (dependent variable; *y*-axis), interpreted as the probability of specific environmental combinations to mirror the conditions where the species actually occurs. Correlative ecological niche model (ENM) can also be based on logistic regression based on relation between continuous environmental variables and binary reports of the species (i.e., presence/absence). Note that the descriptive model (red line) is evaluated in terms of its capacity to accurately resemble the data; the information lost is expressed as the distance between the model and the data employed for model calibration (right). **(B)** Predictive model (same as above) intended to forecast the response of the system to an unknown status. Predictive models are generally evaluated based on their capacity to predict independent data (i.e., data not used during model calibration). Note that a predictive model could be simple (red straight line) and could result in the loss of more information. Nevertheless, independent data (red points) may be accurately predicted.

Descriptive models are generally evaluated in terms of the capacity of the model to accurately reconstruct patterns found in the available data. Thus, evaluation metrics used to differentiate between good and bad descriptive models generally account for the amount of information lost (e.g., Akaike's information criterion) (19) (Figure 1A). Predictive models are evaluated based on their capacities to accurately predict, better than by random, new data (i.e., independent data not used during model calibration). Therefore, evaluation metrics used to differentiate between good and bad predictive models commonly measure model capacity to differentiation between actual data and random observations (e.g., *p*-value, sensitivity vs. specificity) (Figure 1B).

Models can also be differentiated based on their capacities of interpolation and extrapolation. Interpolation is defined as an estimation of unknown values present within the range of values from the data used to calibrate the model (20). Extrapolation is defined as the estimation of unknown values beyond the range of data used for calibration. Ideally, models aiming to be descriptive should have low interpolation and extrapolation abilities resembling good fit to the data. Predictive models are expected to interpolate and extrapolate. As a result, the final goal of the model, descriptive or predictive, should guide the design of its calibration and evaluation protocols. A perilous arena within spatial epidemiology is the development of predictive models that are evaluated using metrics developed for descriptive models or that are penalized based on extrapolation (21). Similarly, robust model evaluation of predictive models would require evaluation data statistically independent from calibration data. Thus, data-patitioning methods that do not ensure independency (e.g., cross-validation) have questionable capacity to differentiate between good and bad predictive models.

## Ecological Niche Models

Previous applications of ecology to map infectious disease risk have resulted in successful disease control and prevention [e.g., (22)]. During the last two decades, valuable advancements in alternative approaches to investigate infectious diseases through applied ecology have been made (15). The ability to determine why a disease is present in one animal species, season, and geographic area but absent in others facilitates the understanding of spread and persistence of infectious diseases in wildlife and domestic animal populations, critical for veterinary medicine.

The final goal of ecological niche modeling applications in spatial epidemiology is to determine environmental conditions associated with disease occurrence. This in turn can help to identify localities where such conditions exist and that are suitable for disease introduction, maintenance, and posterior spread. These models can be conducted at the local level using accurate disease reports coupled with landscape information or at the regional level coupled with climatic variables.

Disease distributions at coarse scales are often manifested through climatic variables (e.g., temperature and precipitation) falling across expected ranges of climate values observed in the bulk of confirmed disease reports. In ecological niche theory, the fundamental niche, **N**_*F*_, represents the set of abiotic environmental conditions necessary for long-term population persistence. More specifically, **N**_*F*_ allows population permanence without subsidy from immigration. Variables used to estimate **N**_*F*_ are not modified by the presence or abundance of the organism (e.g., temperature, precipitation) (14). **N**_*F*_ models are usually estimated at coarse-scale based on climatic signatures of biological systems to reconstruct the potential geographic distribution of organisms, revealing areas with suitable climatic conditions across broad regions (14).

**Figure 2:**
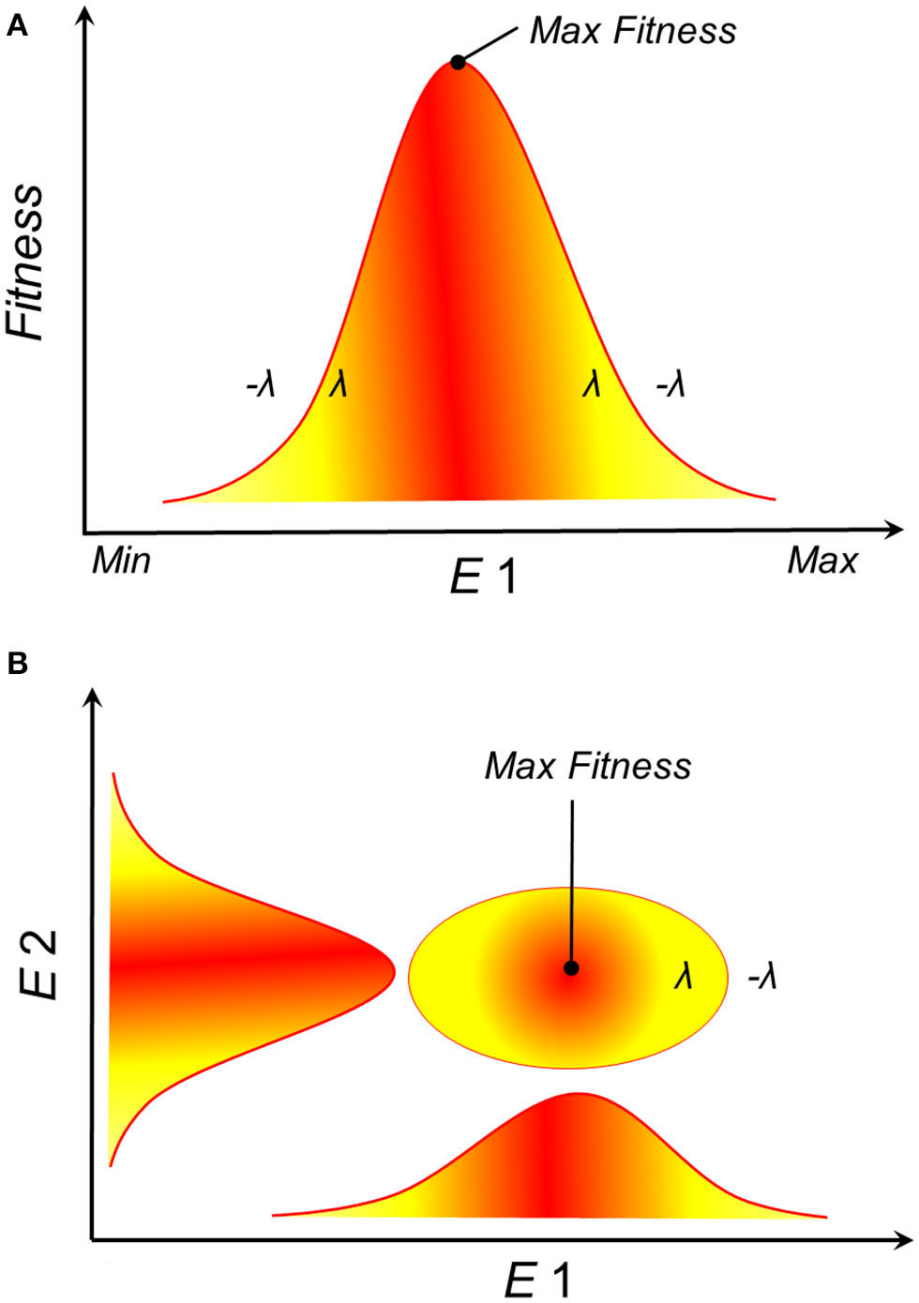
Theoretical representation of the fundamental ecological niche. **(A)** Survival or population growth (*y*-axis), as a proxy of suitability, shows a Gaussian response to environmental gradients (*E*1, *x*-axis). For example, population growth (**λ**) is positive within the environmental range tolerated by the species and negative below or above such range (–**λ**), with an optimum observed at intermediate environmental values (red). **(B)** Ellipsoidal shapes are the expected species response to *n*-dimensional environmental gradients. Example in a bidimensional environmental space where red is higher suitability.

Empirical and theoretical evidence from physiological experiments suggests that population growth and survival of species often have a Gaussian response to environmental gradients (23–28). That is, theory suggests that an organism's fitness responds to environmental conditions with a normal curve, where extremely low and extremely high environmental values drive low fitness, while intermediate environmental values are the optimum for fitness (Figure 2A). **N**_*F*_ accounts for multiple environmental variables, and when many environmental variables are considered, each with a Gaussian response curve, their combination could resemble an ellipsoid (Figure 2B). Consequently, Maguire proposed that the **N**_*F*_ should be convex in shape (25), with ellipsoids offering simple proxies of such convex shape (29). The Gaussian response of organisms to environmental conditions suggests that a disease reservoir or vector would have varied demographic parameters in different sections of its **N**_*F*_. As a result, disease control on reservoirs or vectors (e.g., culling, vaccination) could have different effects under alternative environmental conditions. More specifically, this theory suggests that highest transmission should be expected in the optimal environmental conditions suitable for a disease reservoir or vector (30) and population health management decisions should be made accordingly.

Nevertheless, all the environmental conditions in **N**_*F*_ may not be entirely available for the species. Thus, **N**_*F*_ is hard to reconstruct with field data due to its theoretical nature; however, other portions of it may be more feasible to estimate. The realized niche, **N**_*R*_, is the portion of the **N**_*F*_ that is actually occupied by the organism, reflecting fine-scale constraining effects of dispersal limitations and biotic interactions (14). Fine-scale ecological niche modeling is generally achieved by linking landscape-level variables (e.g., vegetation, host density) as a proxy of the **N**_*R*_. Essentially, **N**_*R*_ is a close representation of the actual conditions present across the distribution of an organism. Recent experimental research shows that landscape materials (e.g., grass, wood, soil, and water) can play a role in the maintenance and spread of pathogens facilitating environmental transmission (31, 32). Satellite-derived data of vegetation phenology, soil composition, moisture, and microclimate have served as proxies of landscape features, allowing researchers to capture environmental signatures of pathogen distribution at the local level (6, 33).

**Figure 3:**
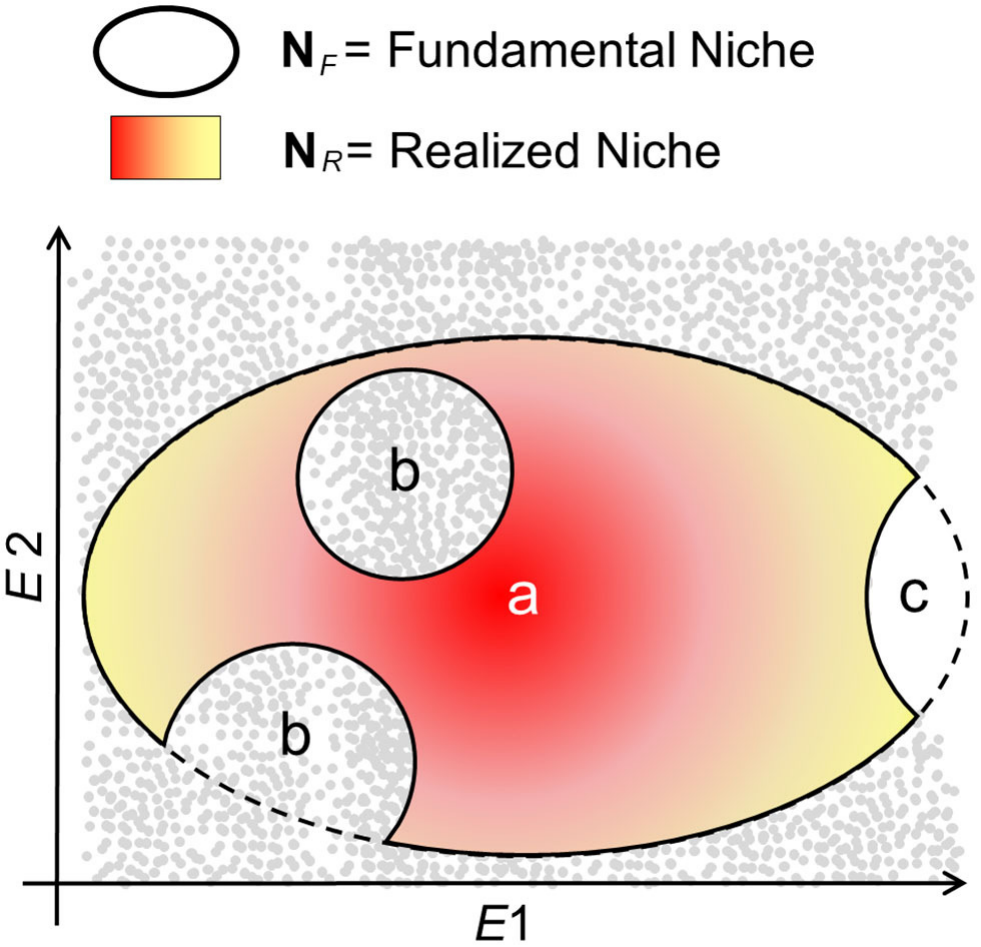
Schematic of N_*R*_ vs. N_*F*_ in a disease system. The fundamental niche, **N**_*F*_, denotes the environmental conditions, available (gray points) or not (white), that are suitable for the organism (e.g., pathogen, vector, and reservoir) to persist and establish populations in the long term. **N**_*F*_ is represented as an ellipsoid with *a* denoting the theoretically most suitable conditions (dark red), with suitability declining to the edges of **N**_*F*_ (yellow). The realized niche, **N**_*R*_, denotes the environments in **N**_*F*_ actually occupied by the organism. Nevertheless, **N**_*R*_ may be a subset of **N**_*F*_ maybe because the organism does not occupy some environments due to dispersal limitations (*b*), because there is not host of vector for the organism's persistence, or because the suitable environmental conditions do not exist in the area accessible (*c*), reducing **N**_*F*_ to **N**_F_*.

Ecological niche models assume that the biotic interactions restrict species to occupy their entire **N**_*F*_; therefore, that **N**_*R*_ is a portion of **N**_*F*_ (14, 34) (Figure 3). That is, maybe not all the utilizable conditions in the pathogen's **N**_*F*_ have the presence of the host. Additionally, ecological niche inferences must consider the geographic area accessible to the organism (termed **M**) and the set of environments represented across that region (14) as the limits of **N**_*F*_ that are available. That is, maybe not all the climates utilizable by the organism exist in the areas of study so that the existing fundamental niche, ${\text{N}}_{F}^{*}$, represents the portion of **N**_*F*_ that the species could use. ${\text{N}}_{F}^{*}$ is the intersection of **N**_*F*_ with the area accessible **M**, such that the existing ${\text{N}}_{F}^{*}$ will be a subset of **N**_*F*_; any attempt to use the existing ${\text{N}}_{F}^{*}$ or **N**_*R*_ as estimates of **N**_*F*_ is perilous for species with limited dispersal (35). Based on this reasoning, the selection of the study area has dramatic implications on the environmental conditions to be modeled and, in turn, on estimations of **N**_*F*_ or **N**_*R*_.

## The Problem of Scale in Infectious Disease Ecology

**Figure 4:**
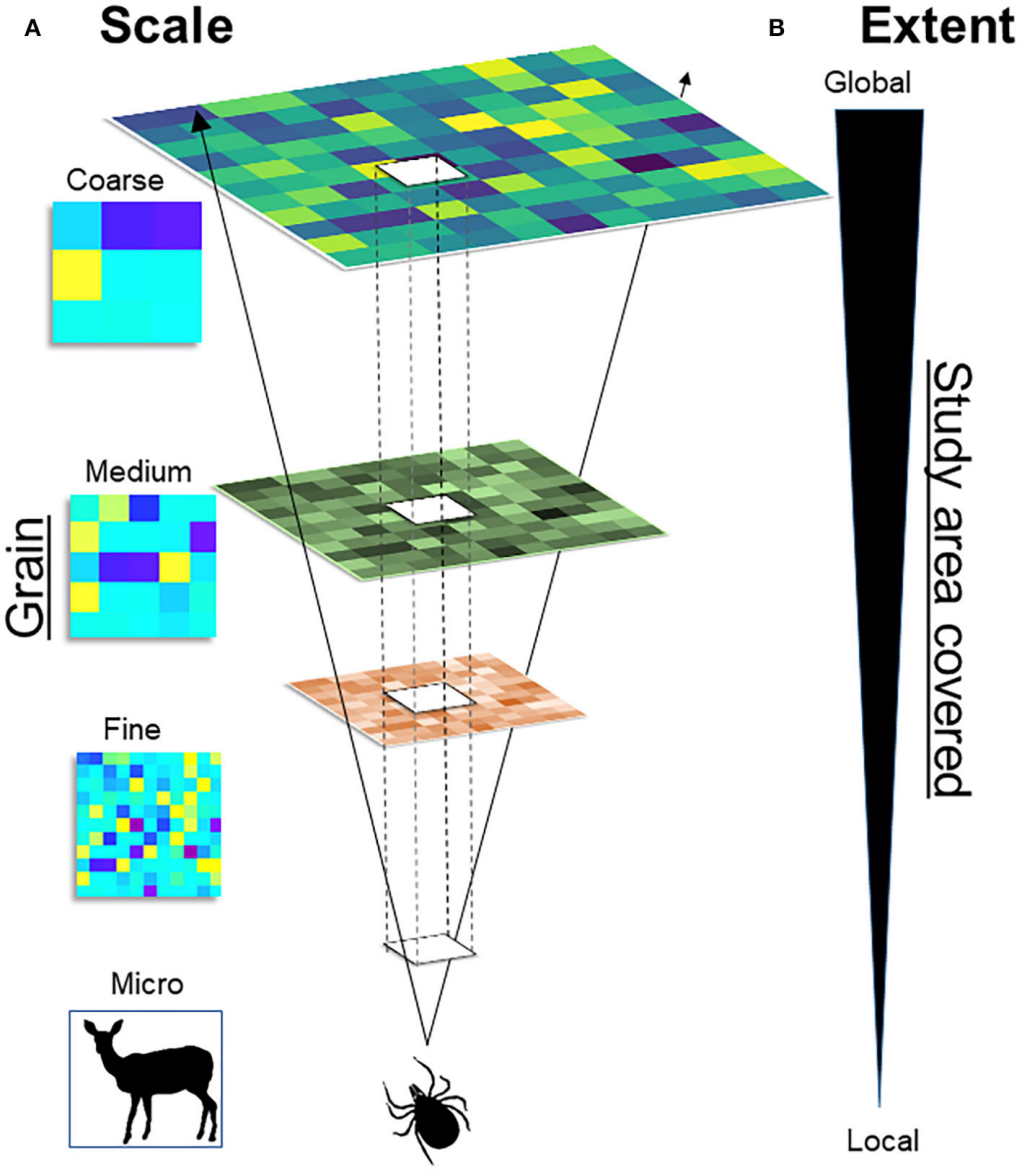
Multiscale framework. **(A)** Scale: Scale of variables (i.e., temporal and spatial) varies from the micro, to the fine, to the coarse. For example, ticks could be studied at the micro scale, by assessing its distribution across the skin of the host (e.g., deer). **(B)** Extent: Represents the size of the study area, from the local to the global extent. For example, increasing the extent will allow to study ticks across a forest (local) or across a continent (global). The scale and the extent are correlated: Fine-scale studies provide high detail (fine grain) but cover small study areas, coarse-scale studies cover large areas at the cost of detail (large grain).

A major challenge in spatial epidemiology and distributional ecology is the identification of the scale of the analysis for a statistically correct study design and a biologically sound model interpretation. While this question may appear to be easily answered based on the data available, generally, incorrect identification of the scale may result in misleading study designs, misinterpretation of results, and inability to fill primary gaps of knowledge. Studies must identify the temporal scale (from hours and days to decades and millennia, including past, present, and future time) and the spatial scale (from centimeters to kilometers) of interest. For example, studies could be conducted in a protected area during a season, or at the continental level across a 60-years period. It is also important to define the organismal level (from molecules and genes to populations and biomes) upon which the study is focused (36). Because infectious diseases can be examined on a wide scale, from micro to macro, assumptions, data, and model interpretation will vary across scales (Figure 4).

A main assumption in epidemiology is that diseases do not occur randomly, which can be used to assess the distribution of pathogens across taxa and geographies to identify specific patterns that can be modeled and predicted. At the fine scale, models can estimate the likelihood that specific wildlife species will be suitable for vector infestation. At the medium scale, models can assess the landscape drivers of disease transmission. At the coarse scale, models could be used to reconstruct spatial patterns of the extent, direction, and speed of disease spread across continents (Figure 4).

**Figure 5:**
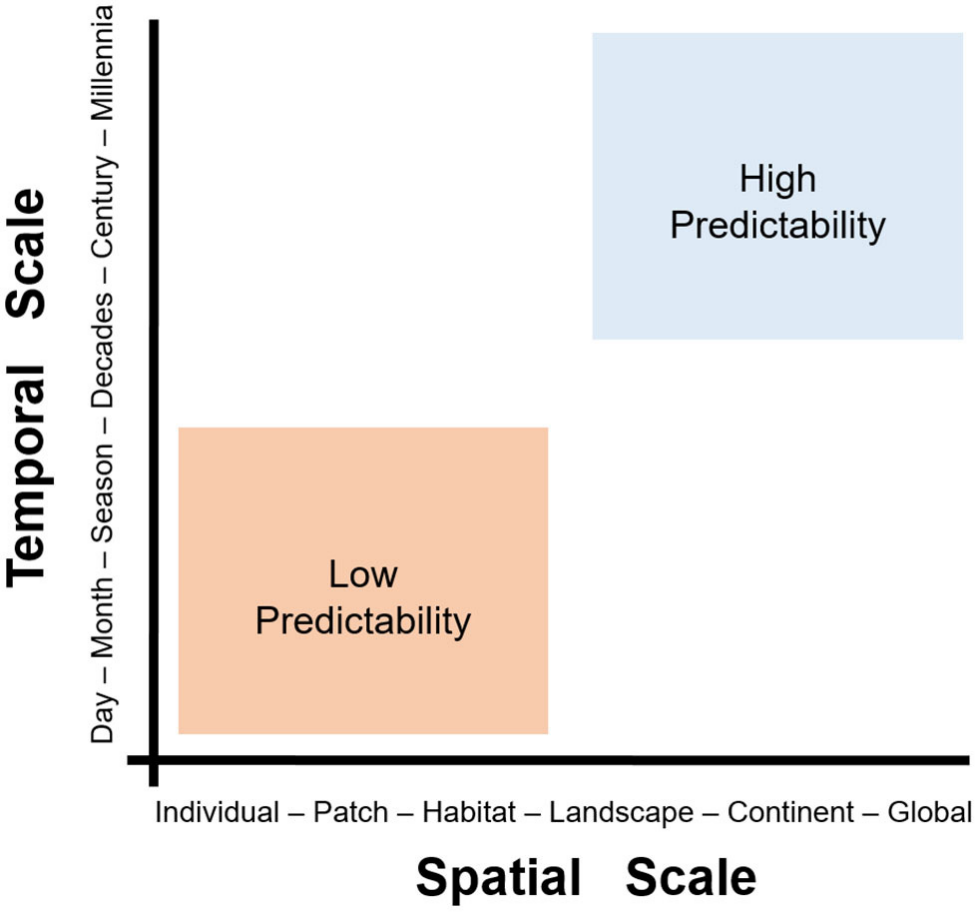
The problem of scale in disease ecology. Scale can be defined in two dimensions: spatial (*x*-axis) and temporal (*y*-axis). Fine-scale studies (e.g., data from at the individual-level during hours to days) can provide high-quality data and a good understanding of phenomena. The figure shows a pragmatic categorization of two spatiotemporal scales, fine (orange) and coarse (blue). Fine-scale studies (orange) tend to offer limited predictability but high detail, while coarse-scale studies (blue) offer high predictive potential at the cost of losing detail. Note that landscape/seasonal studies are somewhere in-between fine- and coarse-scale studies. Modified from Levin (36).

Fine-scale studies are conducted locally and capture individual-level details in short periods of time (e.g., a season). The resources and effort necessary to conduct fine-scale research restrict their development to small study areas (e.g., a forest). Coarse-scale studies, however, can be conducted at large extents but generally fail to capture the details necessary to understand local-level phenomena. The level of detail or grain of variables quantified is linked to the scale, extent, and their capacity of prediction. Coarse-scale studies may lack details but would provide predictions that are more robust across space and time. Thus, the problem of scale in disease ecology is how predictions change as scales change (Figure 5). The problem of scale (i.e., temporal or spatial) has been described in detail by Simon A. Levin (36) and provides opportunities to better understand disease systems across space and time. Interestingly, spatial epidemiology of animal diseases seems to be biased toward local-level studies, with limited research conducted at coarser scales (37).

The organismal level is a major challenge in spatial epidemiology. For instance, the complex transmission cycles of vector-borne, water-borne, or directly transmitted diseases require two (e.g., pathogen and host) or more (e.g., pathogen, multiple vectors, multiple reservoirs, or hosts) species to be included in the model. Often in practice, modelers use a single organism to reconstruct areas of transmission, which could focus on the vector or the pathogen. While a parsimonious approach, it requires a strong understanding of the ecology of the disease in question to identify the organism that best explains the disease system. Thus, a next frontier in ecological niche modeling applications to disease systems is the inclusion of more biological components of the cycle of transmission in the modeling process.

## Ecological Niche Modeling and Spatial Epidemiology

Ecological niche modeling has proven to be a useful tool for forecasting distributions and distributional changes for a vast number of organisms (22, 38, 39) and is increasingly employed to predict distributions of pathogens on diverse spatial scales (15). Traditional ecological niche modeling frameworks, however, may make unrealistic assumptions and therefore yield inaccurate predictions. These modeling frameworks must therefore be revised and amended if they are to work in epidemiology (40). Ecological niche modeling estimates ecological niches of species by linking spatial occurrence records with environmental covariates, *via* correlative or mechanistic approaches (41, 42). Theory and analytical approaches of ecological niches have been described during the last century (43), especially for biodiversity and conservation studies.

A decade ago, it was hypothesized that coarse-scale geographic distributions of species were constrained principally by abiotic environmental conditions (i.e., inert variables) across relevant regions, with biotic interactions having negligible effect [termed the *Eltonian Noise Hypothesis* (14, 29)]. As a result, most modelers have considered it reasonable to assume that influences of biotic interactions could be neglected in ecological niche modeling (29). The Eltonian Noise Hypothesis, however, was conceived in the context of free-living organisms (e.g., plants, birds). Currently, most ecological niche modeling applications do not include biotic variables (i.e., derived from living organisms). Epidemiologists of infectious disease and veterinarians have a clear understanding of the major flaws of models neglecting biotic interactions because infectious diseases are by definition biotic interactions.

Developing models based solely on abiotic variables make model outputs of easy interpretation. For example, a model based on pH and humidity could generate estimates of suitability with regard to environmental conditions. Nevertheless, the role of biotic variables has not been assessed rigorously in parallel analyses in disease ecology [but see (44)]. Indeed, incorporation of biotic variables in ecological niche modeling analyses for diseases was proposed only relatively recently (45), and such applications remain rare in spatial epidemiology. The inclusion criteria of the biotic variables to be used, their temporal and spatial scales, and whether biotic variables should be used before, during, or after the model calibration process remain understudied (40). Currently, use of abiotic-only predictors (e.g., climate) dominates the literature regarding modeling and predicting geographic distributions of pathogens. Including biotic variables in the ecological niche modeling process would require a detailed and *a priori* definition of the modeling outputs. For example, a model including host density or percentage of vaccination coverage would require a revision of the “suitability” term in the context of each study (e.g., suitability for transmission or exposure); alternatively, other terms would need to be employed for modeling disease systems, such as risk (46) or relative occurrence rate (47). Understanding the role of biotic variables in ecological niche modeling may revolutionize the utility of these tools drawn from ecology for disease risk mapping.

## Ingredients to Make an Ecological Niche Model

**Figure 6:**
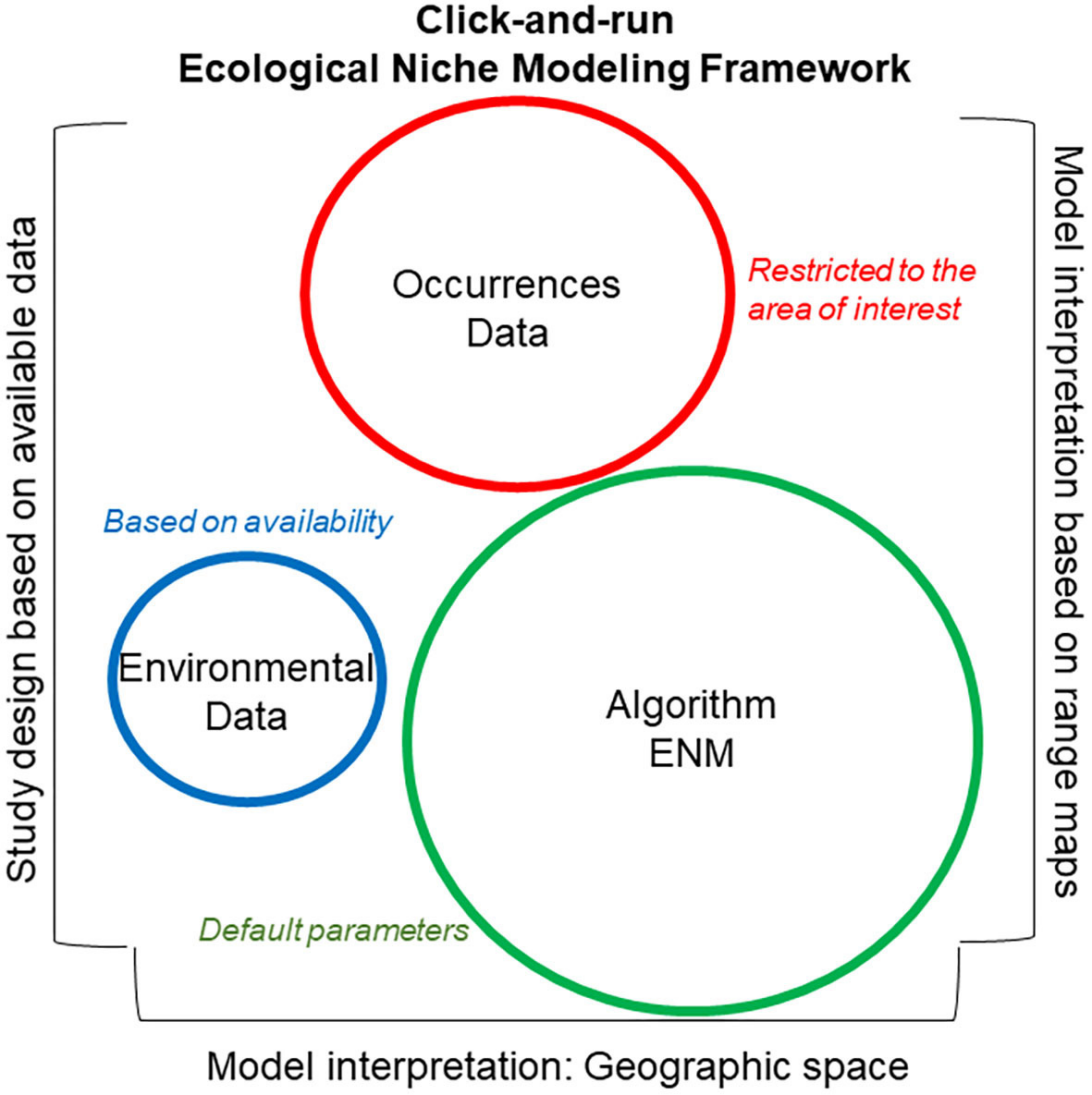
Click-and-run ecological niche modeling framework. This modeling framework is data-driven, with uncaring study designs. Syndromes used in identifying click-and-run ecological niche models include (i) strong emphasis on the good performance of the algorithm employed based on external literature and use of default parameters (green), (ii) moderate emphasis on the quality and quantity of the disease localities (i.e., occurrence data; red), with limited data curation and evaluation, and (iii) little or no justification and defense of the environmental variables selected for model calibration (blue). Additionally, these models generally fail to define the ecological niche to be estimated, **N**_*F*_ vs. **N**_*R*_. The study area of these models is generally delimited based on data availability or administrative boundaries, while model interpretation is generally based on visual inspection of maps. Circle size denotes magnitude of importance assigned to each category.

Historically, “ingredients” to build ecological niche models have been summarized in three major categories: occurrence data, environmental data (abiotic or biotic variables), and algorithm (Figure 6). Occurrence data are represented as disease cases, or serology or direct detection of pathogens or parasites, or records of vectors, intermediate hosts, or reservoirs recorded geographically as coordinates (i.e., latitude and longitude). Environmental data are represented at coarse (i.e., climate) and fine (e.g., vegetation indices) resolutions in terms of the abiotic and biotic environmental conditions where occurrence data are collected. Then, to link environmental conditions and disease occurrence, correlative or classification algorithms are generally used. This analytical framework has been criticized due to the limited understanding of the user regarding the potential data and algorithm limitations, and the theoretical bases of the algorithm employed (48). Careless applications of this simple modeling framework has been termed “click-and-run ecological niche modeling” (49) and has resulted in misleading ecological niche modeling applications (50–52). Indeed, studies to reconstruct disease distributions should avoid using protocols and parameterization scenarios developed for other taxa, regions, or periods. Instead, the modeling protocol for disease mapping should be specific to the study question, data available, and assumptions of the disease system.

The click-and-run modeling framework (Figure 6) is based on the use of “recipes” to model the distribution of any species, neglecting the biology of the organism in question. These models also neglect biases or artifacts in the data used for model calibration and the functionality of the algorithm employed. This approach requires limited data curation and model parameterization and was used in the past for single-species ecological niche models, but is currently used for studies modeling hundreds or thousands of species to capture coarse ecological patterns (i.e., macroecology). Model evaluation in click-and-run modeling is generally poor or absent, making this modeling framework particularly questionable when modeling infectious diseases.

Models should include occurrence data curated carefully to include only trusted occurrence records for model calibration. Trustworthy disease occurrence records should have traceable diagnostic methods, data sources, transparent surveillance protocols, temporal details, and quantified uncertainty (e.g., spatially error, sensitivity of the diagnostic method). That is, selection of the occurrence data should include an exhaustive inspection of the metadata to reduce errors followed by estimations and mitigations of duplicates, autocorrelation, and sampling bias, supported by detailed protocols as described by Cobos et al. (53).

Ecological niche modeling of disease systems should consider abiotic environmental variables (e.g., temperature, soil, precipitation) that fit the scale of interest and the biology of the disease to be modeled. For example, historical satellite-derived bioclimatic data can be employed at a pixel resolution of 20 km, if this is in agreement with the approximate home range size of a pathogen's reservoir (~10 km) (10). In this case, one could use land surface temperature (°C) and ground humidity (kg) to overcome limitations of interpolated climatic data (8). That is, it has been found that satellite-derived data overcome limitations of the original interpolations found in the climatic data from ground stations (54). For example, WorldClim, a commonly used resource for climate data, often includes only 0.001% of empirical data and 99.99% of interpolated data, resulting in high spatial lag (autocorrelation) and frequent aberrant and unrealistic climatic values (15).

Biotic variables (e.g., host density, prey density, and predator occupancy) are biological factors shaping the distribution of a species or disease at the local level (55, 56). Biotic variables must be selected on the basis of the natural history of the target pathogen, including the presence or abundance of other organisms that facilitate or limit its presence. For some pathogens, relevant positive biotic factors that facilitate the presence of disease may include co-infections with other pathogens, vegetation preferences of vectors, and host availability, behavior, and density. Negative biotic factors that limit pathogen circulation and establishment may include host immunity and biodiversity values (40, 46, 57). Biotic components may be critical to understand the ecology of pathogen transmission, and their effects are evident when developing studies at fine geographic scales (46), although the question of their action across broad geographic extents remains unanswered. Nevertheless, based on the biology of the pathogen, biotic variables could include proxies of host availability (58), anthropogenic disturbance (59), wildlife reservoirs availability (60), and barriers of disease spread (61, 62). Each of these dimensions has been found to be predictors of infectious diseases (10, 45, 63–67).

The inclusion of biotic variables in ecological niche modeling could be done before, during, and after the calibration of the model. For example, biotic variables could be used before the development of the model by restricting the distribution of the focal species (e.g., pathogen) to regions where biotic interactions may occur (e.g., host distribution; pre-processing). Biotic variables could be added to model calibration by incorporating biotic factors as predictor variables in the ecological niche modeling (e.g., host density; processing). Alternatively, biotic variables could be used once the model is developed by incorporating biotic variables on the final model output (post-processing). For example, a hypothetical model to estimate transmission risk of rabies (*Lyssavirus*) transmitted by vampire bats (*Desmodus rotundus*) at the local level could include the use of abiotic (e.g., temperature and precipitation) and biotic variables. Biotic variables could include livestock densities as proxy for food resources for the vampire bats (58), surface of roads as proxy of local-scale barriers (61, 62), and satellite-derived nighttime light surface as proxy of populated centers (59), since these variables have been proposed as predictors of rabies in wildlife (10, 63–67). That is, when biotic variables are included to reconstruct a disease system, it is crucial to identify key factors that directly facilitate or limit transmission. Using biotic variables in ecological niche modeling is still not a common practice and more research is necessary in this area to develop a revised modeling framework.

**Figure 7:**
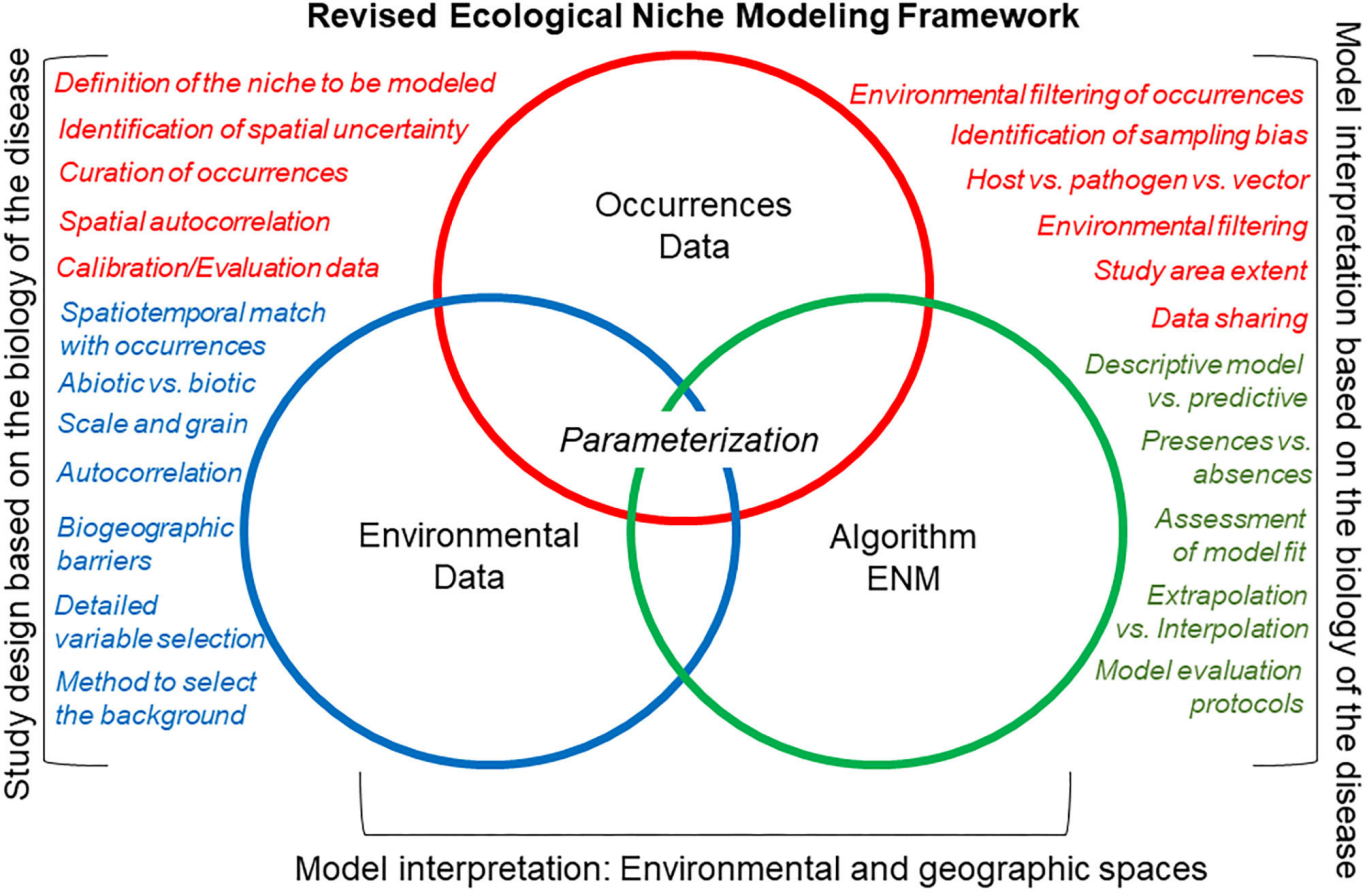
Canonical ecological niche modeling framework (a.k.a. “Peterson's Framework”). This revised ecological niche modeling framework includes some of the critical decisions necessary to develop a comprehensive, replicable ecological niche model that accounts for the natural history of the organism, the quality of the data, and the goal of the model. Here, occurrence data (red), environmental data (green), and the integrative algorithm (blue) have comparable importance regarding their assessment during the modeling process (i.e., same size of circles). The main differences of this canonical ecological niche modeling framework, as compared with the classic ecological niche modeling framework (Figure 6), are that more details are evaluated for each modeling component (circles), model design, and interpretation account for the ecology of the organism and data available, and that model outputs are interpreted in geographic and environmental dimensions.

A revised ecological niche modeling framework could facilitate replicable estimations for any disease system (Figure 7). Nevertheless, each component of a revised modeling framework (i.e., occurrences, environmental variables, and modeling algorithm) would require careful inspection to discard noise signals due to incorrect study designs. That is, study designs should be based on biologically justifiable study areas and variables, which are important drivers of ecological niche modeling performance. In some situations, the protocol will allow one to determine if a robust ecological niche model is feasible or not.

An important component of the revised protocol is the careful inspection of occurrences using a specified inclusion criterion that prioritizes quality over quantity. To assess and mitigate sampling bias in disease reports, modelers can use the method proposed by Varela et al. (68), which compares models from different occurrences filtering methods to mitigate both oversampled areas and oversampled environments. This approach allows the generation of a series of models under different bias mitigation scenarios to (i) reduce model overfitting (i.e., models mirroring closely the data, resulting on limited learning from the model compared with the raw data) and (ii) capture variability for more informed model interpretations. This methodology has been employed broadly to study the distribution of biodiversity but has been barely used to model infectious diseases. In the revised protocol (Figure 7), model calibration could include biotic variables as predictor (45, 69). Nevertheless, researchers must clarify the units and interpretation of the modeling output.

Finally, the study area of interest [**M** *sensu* (70)] is a major component of the modeling process. A common failure in ecological niche modeling applications based on correlative models is to pragmatically determine the study area. Restricting models based on administrative areas (e.g., municipality, department, province, and state) does not account for the biology of the organism. Pathogens do not know about political borders; therefore, models should account for biogeographic barriers (e.g., rivers, roads, impervious surfaces, and oceans) for biologically sound study designs.

**Figure 8:**
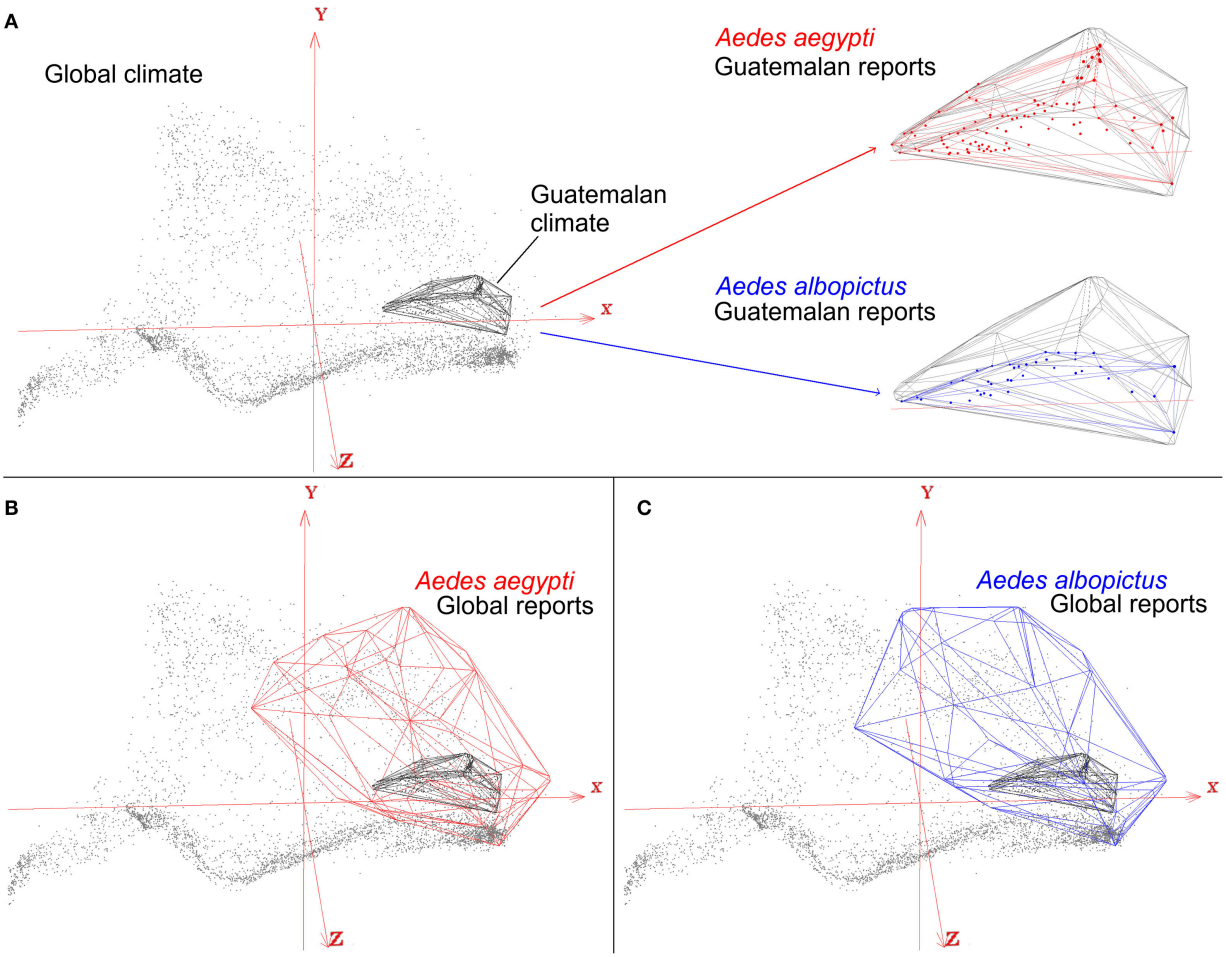
Effects of study area M on ecological niche modeling results. An example of an ecological niche model of arbovirus vectors *Aedes aegypti* (red) and *Ae. albopictus* (blue), which occur worldwide, to predict transmission risk in Guatemala. Global climate data (gray dots) show the broad range and diversity of global environmental conditions summarized in the first three components of bioclimatic variables (red axis). Restricting the study to Guatemala (gray polyhedron) will require to use species records only across this country (red and blue dots), resulting in a subset of occurrence records, and environmental tolerances, of the mosquito species (red and blue polyhedrons) **(A)**. Accounting for all the available data for the vector species, however, reveals that Guatemala only contains a portion of the species environmental tolerances **(B,C)**. As a result, when the study design is restricted to Guatemala as the study area **M**, and to its climate and environmental space, the model would yield in an incomplete reconstruction of the actual distributional potential of vector-borne diseases. Restricting the models to Guatemala would require other, fine-scale, landscape-level environmental variables. Otherwise, models will simply mirror the density of points. Models constructed using an envelope algorithm in NicheA (71).

The perils of careless study-area delimitations will result in models that are misaligned with the primary question of the study, the ecology of the organism, resulting in underestimations of the true potential of the disease spread. For example, the mosquitoes *Aedes aegypti* and *Ae. albopictus* are important vectors of many arboviruses, including Zika, Dengue, and Chikungunya viruses with transmission reported globally. Nevertheless, one may be interested in modeling the distributional ecology of these mosquitoes in a specific study area (Figure 8A). For example, mosquito presence in Guatemala along with climate information from the sampling region will only capture environmental tolerances of the species in that particular area. This may therefore result in a gross underestimation of the true potential distribution of the vectors and the diseases they transmit. Indeed, these mosquito species are ecological generalist species that tolerate a broad range of climatic conditions and have global distributions (Figures 8B,C). Thus, real tolerances and actual potential distribution of species could be masked by a restricted study area that only accounts for a portion of the species truly potential.

## Ecological Niche Modeling Algorithms and Tools

**Figure 9:**
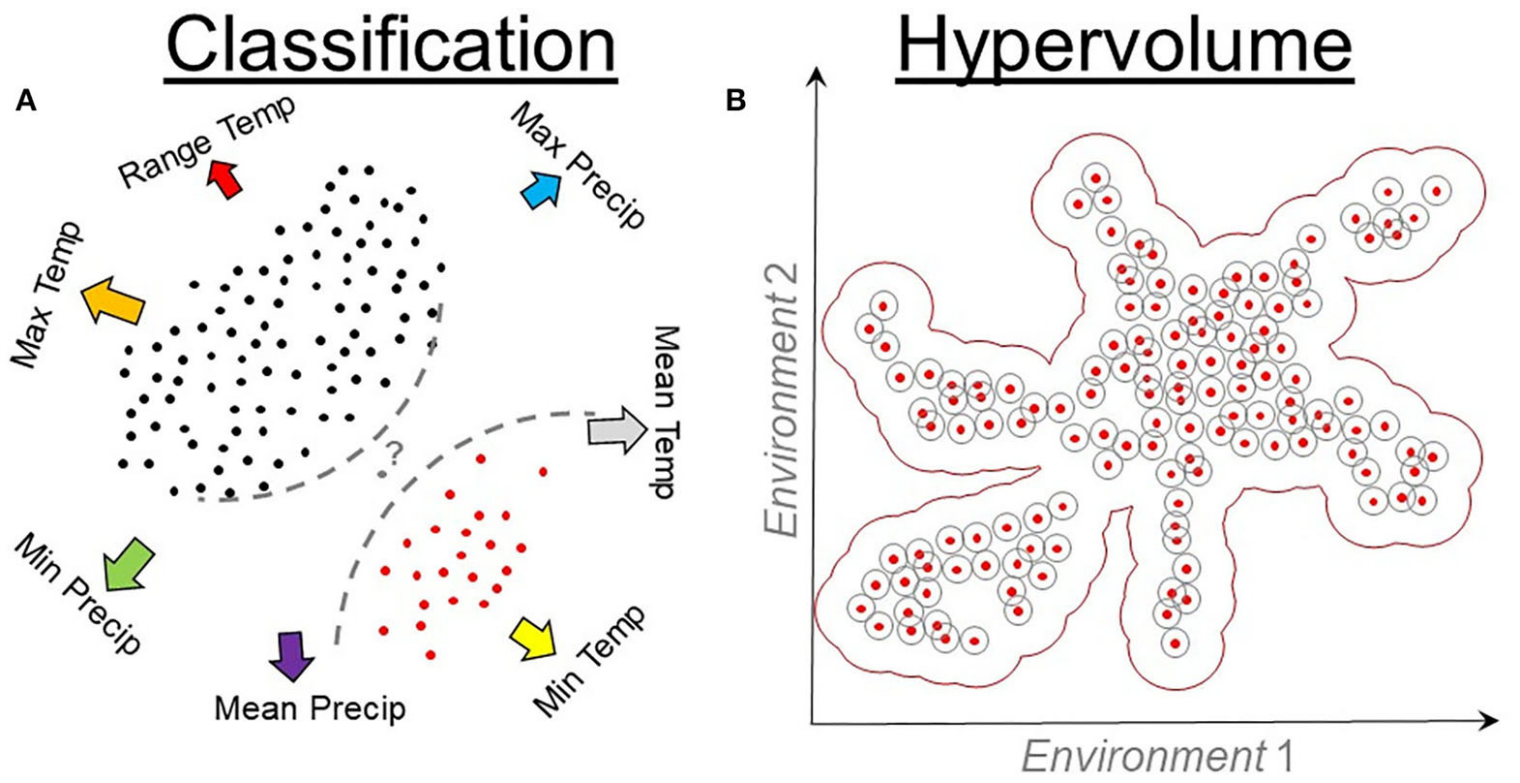
Classification and hypervolume models. **(A)** Classification algorithms require environmental values where the species is present (red points) and absent (black points). Presence and absence data are linked to environmental values (arrows) to quantify the probability (question mark) of identifying a locality (gray point) as a suitable or unsuitable. Classification algorithms use these data to inform a series of rules (dashed lines) that vary among algorithms. Temp, temperature; Precip, precipitation. **(B)** Hypervolume algorithms quantify the density or cluster of presence records of the organisms in environmental dimensions. Hypervolumes measure the distance (gray cycles) among occurrences (red points) in an environmental space (arrows) to determine a best-fit model (red buffer).

Modeling algorithms in ecological niche modeling have been described elsewhere (47, 72–74), generating starting points for new modelers. Algorithms to develop ecological niche models can be divided into three categories: presence-absence, presence-background, and presence-only. Presence-absence algorithms need a set of localities where the organism occurs (i.e., presence) and a set of localities where the organisms does not occur (i.e., absence). Presence-absence models are calibrated by comparing environmental conditions where the organism is present vs. where it is absent and are generally useful to reconstruct the distribution of diseases at fine scale and short periods, resulting in the need of accurate localities and high-resolution environmental variables. These models, however, have limited capacities to be projected to different areas or periods, instead, their signals are space and time specific. Many algorithms are available including regression (e.g., Generalized Linear Models and Generalized Additive Models) (Figure 1) and classification (e.g., Boosted Regression Trees, Random Forest, and Support Vector Machines) (Figure 9A) algorithms, with protocols described in detail elsewhere (75).

Occurrence data are generally robust, while absence data are largely questionable in quality and of limited availability [discussed in (14)]. To solve this problem, researchers generally “simulate” absence data to be able to use presence-absence algorithms. A common approach to simulate absence data is to generate random points across the study area. Presence-absence models that use simulated (i.e., fake) absence data during calibration are termed presence-background models. Presence-background algorithms thus use the same regression and classification algorithms used for presence-absence models, with the unique philosophical variation regarding the interpretation of absences vs. background points. Also, because the background corresponds to the study area, calibration of these algorithms is highly sensitive to variations in the extent of the study area extent selected.

Maxent is a popular ecological niche modeling algorithm based on logistic-like regressions comparing densities of occurrences (presences), densities of random points (background), and continuous environmental variables using diverse sets of parameters in the calibration process (47). Maxent protocols have been summarized in a series of software including *Wallace* (76), *dismo* (75), *ENMeval* (77), and *KUenm* (78) packages in R. Wallace is a user-friendly analytical environment to calibrate Maxent models, making it a good starting point for new users since it contains detailed instructions (76). Dismo provides less details regarding the different assumptions and complementary scientific literature, but it is a good starting point for new users interested on modeling in programming environments (75). ENMeval is essentially the programming environment of Wallace and allows more detailed parameterization and evaluation of models (77). KUenm allows detailed, reproducible ecological niche models using Maxent and provides detailed model calibration and selection not available in the other packages (78), overcoming some of the perils of niche model applications for infectious diseases regarding differentiation between good and bad models (46). The KUenm package would be an ideal choice for advanced users since parameterization and installation would require advanced programming skills.

Presence-only algorithms focus solely on the environmental values linked to each occurrence record for calibration. As a result, calibration of these modes is insensitive to changes in the extent of the study area. Classic presence-only methods include environmental envelopes, which are ellipsoids, squares, or convex-hull that surround the occurrences in an environmental space (Figure 9), with algorithms that include Bioclim (75) and NicheA (71). Emerging presence-only methods include hypervolumes estimated using estimators of density (79) and cluster of occurrences in the environmental space (80). Protocols for hypervolume estimations have been described elsewhere (34, 74), and their use is expected to become common for **N**_***R***_ estimations due to the automatization of their workflows and computational optimization.

## Ecological Niche Modeling and Climate Change

A key set of questions in spatial epidemiology relates to effects of global change on the geographic distribution of infectious diseases and the potential of disease reservoirs or vectors to respond to such changes (81). Global change includes climate and land cover changes and the accelerated introduction of invasive species (82–84). A recent assessment proposed that catastrophic climate change effects will be perceived with even a 1.5°C annual mean temperature increase in the coming decades (85).

Ongoing climate change trends have been defined as human-induced, with unprecedented effects on biodiversity, impacting many organisms involved in disease transmission cycles (86). Climate change in the Anthropocene is generating geographic (87) and elevational (88) shifts of biodiversity, including organisms involved in disease transmission (89). Climate change is expected to produce bigger and more frequent weather events and wildfires (90, 91) and reductions of crop yields (92, 93), which together could generate ecological imbalance facilitating pathogen spillover (94). Understanding climate effects on directly transmitted diseases, however, remains in its infancy. Ecological niche models are a promising tool to help anticipate likely responses of disease systems to climate change. Recent assessments of vector-borne diseases have challenged paradigms related to climate and infectious diseases (95–97).

A recently published meta-analysis demonstrated that many popular algorithms for ecological niche modeling generally overestimate organisms' ability for adaptation to changing environments (98). The best forecasts should come from analyses of extensive data with simple algorithms (21, 99). That is, robust models require abundant, high-quality input occurrence data; these data are generally limited in availability in developing countries, so research about global change effects on diseases may be biased to developed countries (100). Nevertheless, even when data limitations may exist, ecological niche models provide opportunities to understand how global change can affect infectious diseases globally. Based on the observations described above, when limited data are available, the use of multiple algorithms could help to explain uncertainty in model estimates.

The present understanding of potential climate change effects on organisms is biased geographically to temperate-zone countries (101). Nevertheless, tropical countries already show considerable climate change manifested in just the last three decades. Hence, the tropics represent an important priority for global change disease ecology research in view of their considerable research gaps, their role in modulating global climate, the need to understand organisms' responses to environmental change beyond temperate areas, and the need to assess niche evolution empirically in more rigorous analyses (102).

## The Paradox of Directly Transmitted Diseases

It is not surprising that ecological niche modeling applications in spatial epidemiology are biased toward vector-borne diseases. Data of disease vectors (e.g., fleas, mosquitoes, ticks) are broadly and openly available for many diseases and regions (103). Vectors are also highly responsive to changes in microclimate, with strong responses in their abundance, richness, distribution, and behavior linked to climate and landscape variation (104). As a consequence, ecological niche models of vector species provide good proxies of potential distributions of vector-borne diseases.

Environmentally transmitted diseases, such as anthrax, leptospirosis, and histoplasmosis, can also be studied using ecological niche modeling. Key components of the models include variables resembling the environmental drivers of parasite and pathogen persistence in the environment (e.g., humidity, temperature, and soil pH). When such variables are not available, some proxies could be used with their respective caveats.

Ecological niche models have many advantages compared with other disease modeling approaches, especially with regard to the biological bases that support the use of environmental drivers to map disease distributions. Nevertheless, ecological niche models are not suitable for the study of many disease systems, especially for studies aiming to understand direct transmission between individuals or populations. In such situations, other modeling approaches could be more appropriated (e.g., compartmental models). Similarly, ecological niche modeling may be a perilous modeling framework to use for animal disease systems where the environmental conditions are less important for transmission compared with animal density or human behavior.

Directly-transmitted diseases are more challenging to map based on environmental conditions. Many fine-scale factors (e.g., host density, age, immune status) shaping direct disease transmission may be required for correct reconstruction of transmission, but variables of such factors are generally not available. When the directly-transmitted disease includes an animal reservoir (e.g., wildlife), ecological niche models can focus on such species for the reconstruction of likely areas of transmission.

Ecological niche modeling of directly-transmitted animal diseases are a “dark side” that many veterinary epidemiologists avoid. Limited data of crucial factors associated with transmission and potential economic and ethical implications generally reduce explorations of directly-transmitted animal diseases. For example, the porcine reproductive and respiratory syndrome (PRRS, caused by a virus from the family Arteriviridae) affects the pork industry so that understanding and anticipating its distribution may have enormous benefits for its control and prevention in pig farms. Nevertheless, intensive farms may have controlled environmental conditions, so that the environmental conditions inside the pig farms may not reflect the surrounding climatic conditions. Thus, ecological niche modeling of PRRS risk based solely on the surrounding climate of farms is analytically and computationally feasible [e.g., (105, 106)], but such models will provide an erroneous signals of the environmental conditions suitable for transmission. That is, even when one can model linkages between climate and reports of directly-transmitted diseases, such models could be incomplete, biased, or misleading, and local factors may be more important (107). Paradoxically, models of directly-transmitted diseases are still popular.

## Conclusion

Spatial epidemiology of animal diseases seems to be dominated by local-level studies (37). Thus, ecological niche modeling approaches provide an opportunity to reconstruct environmental conditions suitable for diverse animal diseases to identify areas where transmission is expected. Since disease systems need at least two organisms interacting (host and pathogen), biotic interactions may lie at the core of the pathogen's ecological niche, and neglecting interacting organisms in pathogen dynamics (i.e., maintenance, reproduction, transmission, and spread) may limit the success of forecasts. Pathogen transmission is strongly influenced by fine-scale interactions among infected and susceptible hosts, which can be further affected by host behavior and pathogen demography/transmission. Given the complexity of these interactions, traditional single-species ecological niche modeling approaches could fail to predict disease distributions and transmission risk accurately and protocols need to be revised with caution.

A new challenge in veterinary epidemiology is to avoid falling behind advances that distributional ecology offers in terms of theory and methods to map parasites, pathogens, vectors, and reservoir. This overview is by no means a detailed summary of all the advances in the field of ecological niche modeling. Instead, this review provides a brief introduction to the field facilitating a more effective use of the comprehensive ecological niche modeling courses freely available (e.g., https://www.youtube.com/watch?v=vj8qTo56rPA&ab_channel=A.TownsendPeterson) (108). Veterinary epidemiology needs more ecology, and ecologists modeling disease distributions need to incorporate health professionals for sound and biologically realistic model interpretations (15). Veterinary epidemiologists may find ecological niche modeling useful for disease control efforts, especially for infectious diseases with vectors or wildlife reservoirs. The limited presence of epidemiologists and disease ecologists in the ecological niche modeling community increases the risk of inaccurate and misleading forecasting of infectious diseases of questionable quality and usefulness for stakeholders [e.g., (109)]. The broadly available epidemiological data, collected systematically from humans, animals, and plants, can help to advance the study of disease transmission. The comprehensive understanding of disease systems by veterinarians provides unique opportunities for their active participation in the field of spatial epidemiology. Nevertheless, mature and ethical ecological niche modeling applications for disease mapping would require familiarity with classic ecological theory.

## Author Contributions

The author confirms being the sole contributor of this work and has approved it for publication.

## Conflict of Interest

The author declares that the research was conducted in the absence of any commercial or financial relationships that could be construed as a potential conflict of interest. The reviewer GM declared a past co-authorship with the author LE to the handling editor.
